# *In vivo* and *ex vivo* methods of growing a liver bud through tissue connection

**DOI:** 10.1038/s41598-017-14542-2

**Published:** 2017-10-26

**Authors:** Yusuke Yanagi, Koichi Nakayama, Tomoaki Taguchi, Shin Enosawa, Tadashi Tamura, Koichiro Yoshimaru, Toshiharu Matsuura, Makoto Hayashida, Kenichi Kohashi, Yoshinao Oda, Takayoshi Yamaza, Eiji Kobayashi

**Affiliations:** 1 Department of Pediatric Surgery, Reproductive and Developmental Medicine, Kyushu University Graduate School of Medical Sciences, 3-1-1 Maidashi, Higashi-ku, Fukuoka, 812-8582 Japan; 2 Department of Regenerative Medicine and Biomedical Engineering, Faculty of Medicine, Saga University, 1 Honjo-machi, Saga, 840-8502 Japan; 3 Department of Organ Fabrication, Keio University School of Medicine, 35 Shinanomachi, Shinjuku-ku, Tokyo, 160-8582 Japan; 4 Cyfuse Biomedical K.K., 2-27-17Hongo, Bunkyo-ku, Tokyo, 113-0033 Japan; 5 Department of Anatomic Pathology, Pathological Sciences, Kyushu University Graduate School of Medical Sciences, 3-1-1 Maidashi, Higashi-ku, Fukuoka, 812-8582 Japan; 6 Department of Molecular Cell Biology and Oral Anatomy, Kyushu University Graduate School of Dental Science, 3-1-1 Maidashi, Higashi-ku, Fukuoka, 812-8582 Japan

**Keywords:** Regenerative medicine, Hepatocytes

## Abstract

Cell-based therapy has been proposed as an alternative to orthotopic liver transplantation. The novel transplantation of an *in vitro*-generated liver bud might have therapeutic potential. *In vivo* and *ex vivo* methods for growing a liver bud are essential for paving the way for the clinical translation of liver bud transplantation. We herein report a novel transplantation method for liver buds that are grown *in vivo* involving orthotopic transplantation on the transected parenchyma of the liver, which showed long engraftment and marked growth in comparison to heterotopic transplantation. Furthermore, this study demonstrates a method for rapidly fabricating scalable liver-like tissue by fusing hundreds of liver bud-like spheroids using a 3D bioprinter. Its system to fix the shape of the 3D tissue with the needle-array system enabled the fabrication of elaborate geometry and the immediate execution of culture circulation after 3D printing—thereby avoiding an ischemic environment *ex vivo*. The *ex vivo*-fabricated human liver-like tissue exhibited self-tissue organization *ex vivo* and engraftment on the liver of nude rats. These achievements conclusively show both *in vivo* and *ex vivo* methods for growing *in vitro*-generated liver buds. These methods provide a new approach for *in vitro*-generated liver organoids transplantation.

## Introduction

Half a century has passed since the first human liver transplantation was performed. While orthotopic liver transplantation (OLT) has become a definitive treatment for end-stage liver disease, a shortage of organ donors has limited its benefit^1^. Hepatocyte transplantation has been proposed as an alternative to OLT over the past decades. Evidence of the replacement of the liver with suitable cells suggests that cell-based therapy holds the most promise as an alternative to OLT^2,3^. Although an increasing number of clinical trials of hepatocyte transplantation have shown therapeutic effects, low-level engraftment and the lack of long-term efficacy are major obstacles to this treatment^4,5^. The amount of cells that are transplantable via the endovascular transplantation of hepatocytes has been limited due to portal hypertension and embolism, and the direct injection of hepatocytes into blood vessels resulted in the clearance of transplanted cells by the blood flow and evoked an immune reaction^6^. Tissue engineering technologies have been under investigation as an alternative approach to cell-based therapy, and the engineering of liver organoids is currently an emerging area of study in regenerative therapy for the liver^7,8^. A recent study on the transplantation of *in vitro*-generated liver buds demonstrated outstanding therapeutic potential^9^. To increase the utility of liver bud engineering technology, it will be necessary to develop a method for growing a liver bud into a scalable organoid.

**Figure 1:**
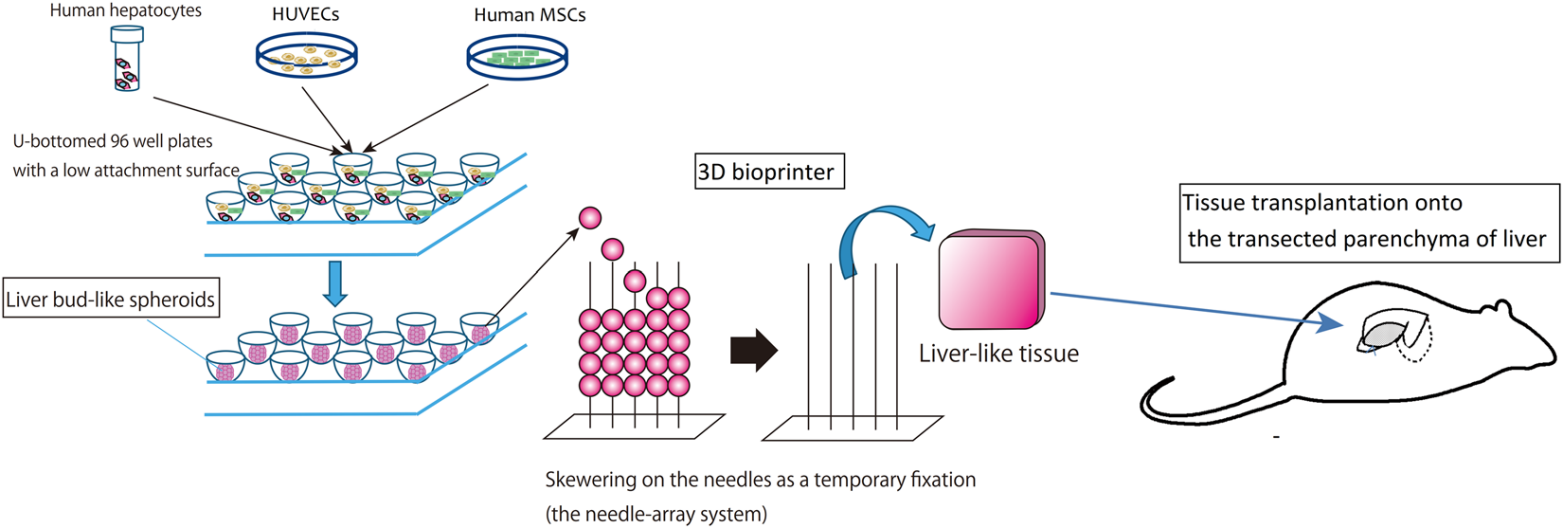
A schematic diagram of our tissue transplantation strategy. Scalable liver-like tissue was constructed using the 3D bioprinter and was transplanted onto the transected parenchyma of the liver.

In the present study, we developed both *in vivo* and *ex vivo* methods for growing liver buds (Fig. 1). We first investigated appropriate sites for the transplantation of liver buds. We hypothesized that although many reports have described the engraftment of liver tissue at ectopic sites^10,11^, orthotopic transplantation would provide a more desirable environment for the growth of the transplanted liver bud in comparison to the environment provided by heterotopic transplantation via direct connection with the recipient’s native liver. The endovascular transplantation of a liver bud is not feasible because vascular embolization occurs due to transplanted cell size-dependent occlusion^12,13^. Thus, we developed a new method for the orthotopic transplantation of liver buds that directly connects the transplant to the recipient’s native liver. This method, which was termed “the orthotopic transplantation on the transected parenchyma of the liver,” resulted in the superior growth of transplanted liver bud in comparison to heterotopic transplantation.

Our next challenge was to develop a method for fabricating a scalable graft from *in vitro*-generated liver buds, which would allow us to transplant a greater number of cells at one time. Recently, various technologies for assembling cellular aggregates to fabricate 3D tissues (with or without a scaffold) have been reported^14,15^. However, these technologies have some disadvantages, including several problems that are associated with the exogenous materials of the scaffold^16^, low physiological strength, and the conditions of tissue culture. To overcome these problems, we used a new 3D bioprinting technology that is capable of rapidly and automatically fabricating a transplantable scaffold-free 3D tissue in an arbitrary shape^17,18^. A liver-like tissue was fabricated without a scaffold by fusing hundreds of liver bud-like spheroids. Finally, the engraftment of the biofabricated liver-like tissue on a rat liver after transplantation using our transplant method was observed to investigate the efficacy of our method. Although a further study to bridge the gap between the real and the engineered liver organoid is necessary, the results of the present study support the idea that orthotopic transplantation provides a hospitable environment for liver tissue engraftment and shows a new approach to cell-based therapy, which may lead to tissue transplantation in the liver.

## Results

### The development of a new method for connecting transplanted liver tissue to the recipient liver parenchyma

**Figure 2:**
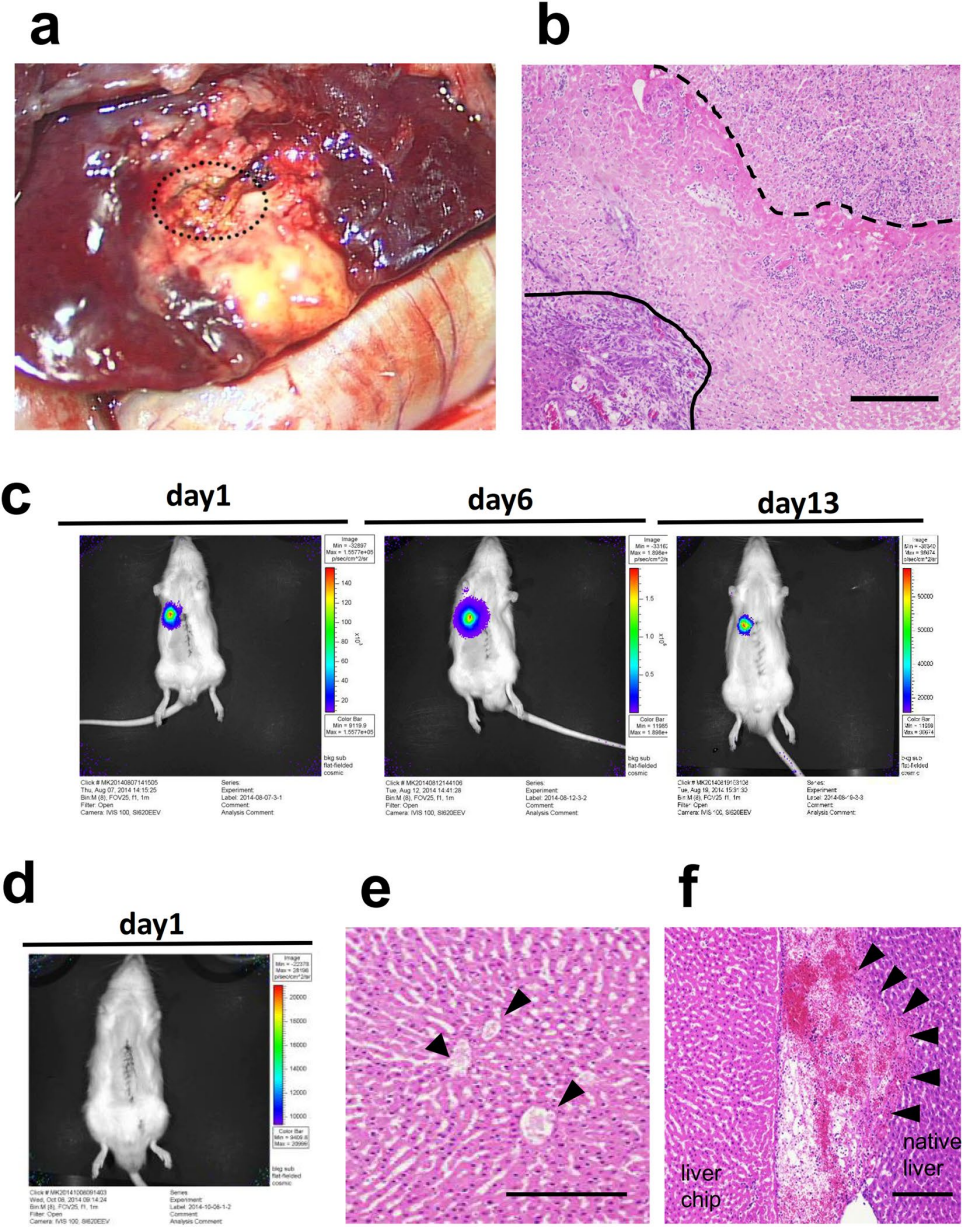
The development of a new method for transplanting liver tissue. (**a**) Gross observation of the native liver that was burned with an ultrasonically activated device. The transplant bed was necrotic and degenerated into yellow tissue. The dashed line indicates the transplanted liver tissue. (**b**) An HE-stained cross-section of the burned site of the native liver. The dashed line indicates the border between the transplanted engineered liver tissue and the burned site of the native liver. The solid line indicates the border between the burned site of the native liver and the normal area of the native liver. Scale bar, 200 μm. (**c**) *In vivo* bioluminescence imaging of a liver chip transplanted onto the transected liver parenchyma showing the engraftment of the liver chip. (**d**) *In vivo* bioluminescence imaging of a liver chip transplanted into a fissure on the surface of liver. The engraftment of the liver chip was not achieved. (**e**) An HE-stained cross-section of the border between the transplanted liver chip. Vascular occlusion was observed (arrowhead). (**f**) The transplanted liver chip was packed with clots. Scale bar, 100 μm in (**b,e,f**).

**Figure 3:**
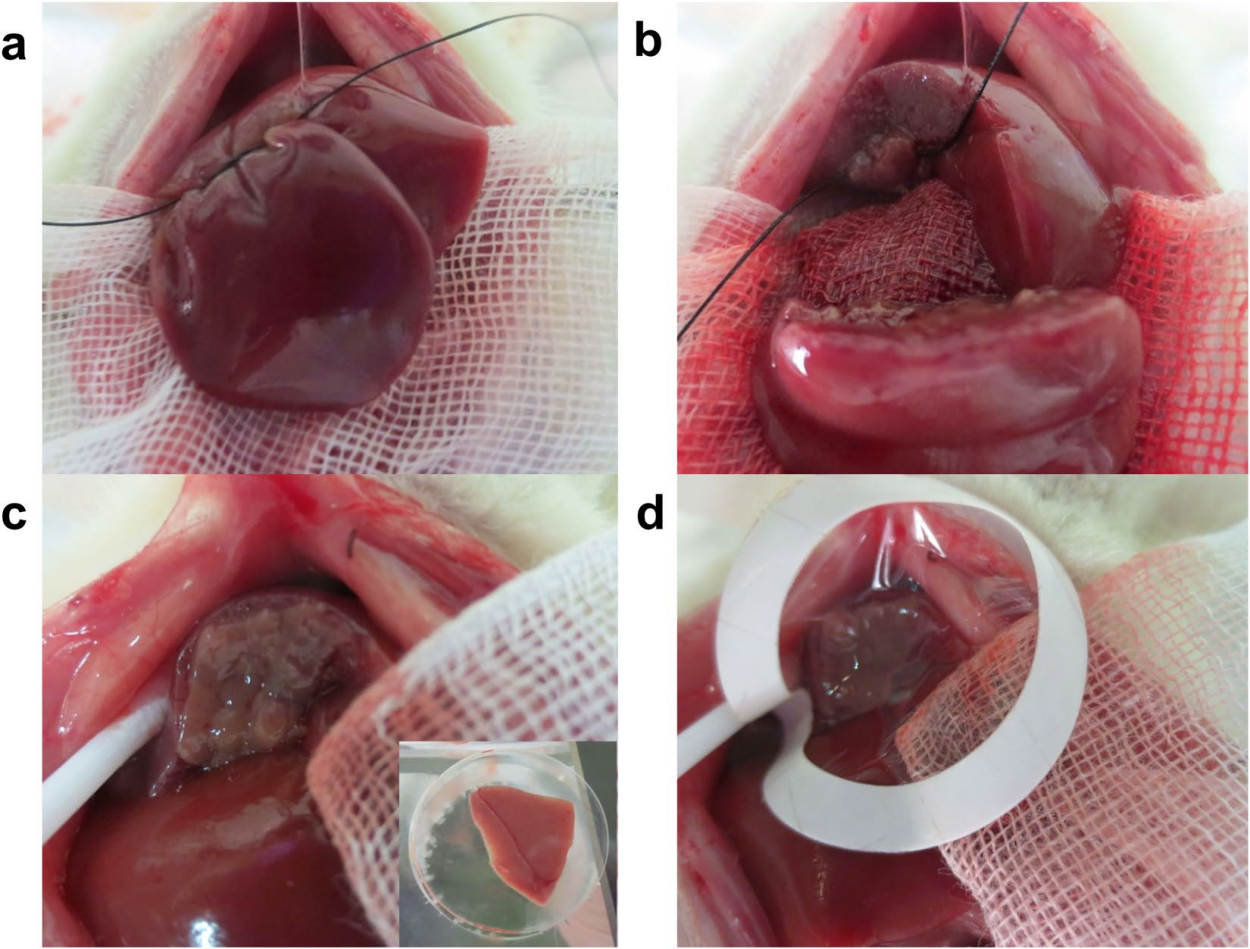
The method for transplanting the tissue onto the transected parenchyma of the recipient rat’s liver. (**a,b**) The liver parenchyma of the median lobe was transected with a surgical suture that was performed with concurrent secure ligation of the vessels to create an intact parenchyma at the transection plane. (**c**) The liver tissue was attached on the transected parenchyma of the median lobe and (**d**) capped with a collagen sheet.

The exposure of the recipient liver parenchyma was necessary in order to connect the transplanted tissue to the recipient liver. Although the liver is prone to bleeding, the use of an ultrasonically activated device to achieve hemostasis burned the liver parenchyma. The hepatocytes in the burned site were not viable and did not accept the transplanted tissue (Fig. 2a,b). This result indicated that an intact liver parenchyma was essential for successful engraftment. Thus, we investigated two methods for creating a transplant site without thermal injury. In the first method, a fissure was created on the liver surface by blunt dissection, and hemostasis was achieved through the application of pressure. In the second method, an intact parenchyma at the transection plane was created by ligating and cutting the parenchyma with a surgical suture; hemostasis was achieved by the concurrent ligation of the vessels (Fig. 3 see also Supplementary Video 1). To compare these two methods, we transplanted a small chip of liver tissue that was harvested from a luciferase transgenic rat^19^ through left lateral lobectomy (30% partial hepatectomy). Transplantation on the transected parenchyma resulted in tissue engraftment (Fig. 2c). In contrast, the intra-liver transplant could not engraft due to vascular occlusion which occurred due to compression and blood packing (Fig. 2d–f). These results indicated that transplantation onto the transected liver parenchyma was a suitable method for transplanting liver tissue.

### The growth of the transplanted liver bud after orthotopic and heterotopic transplantation

**Figure 4:**
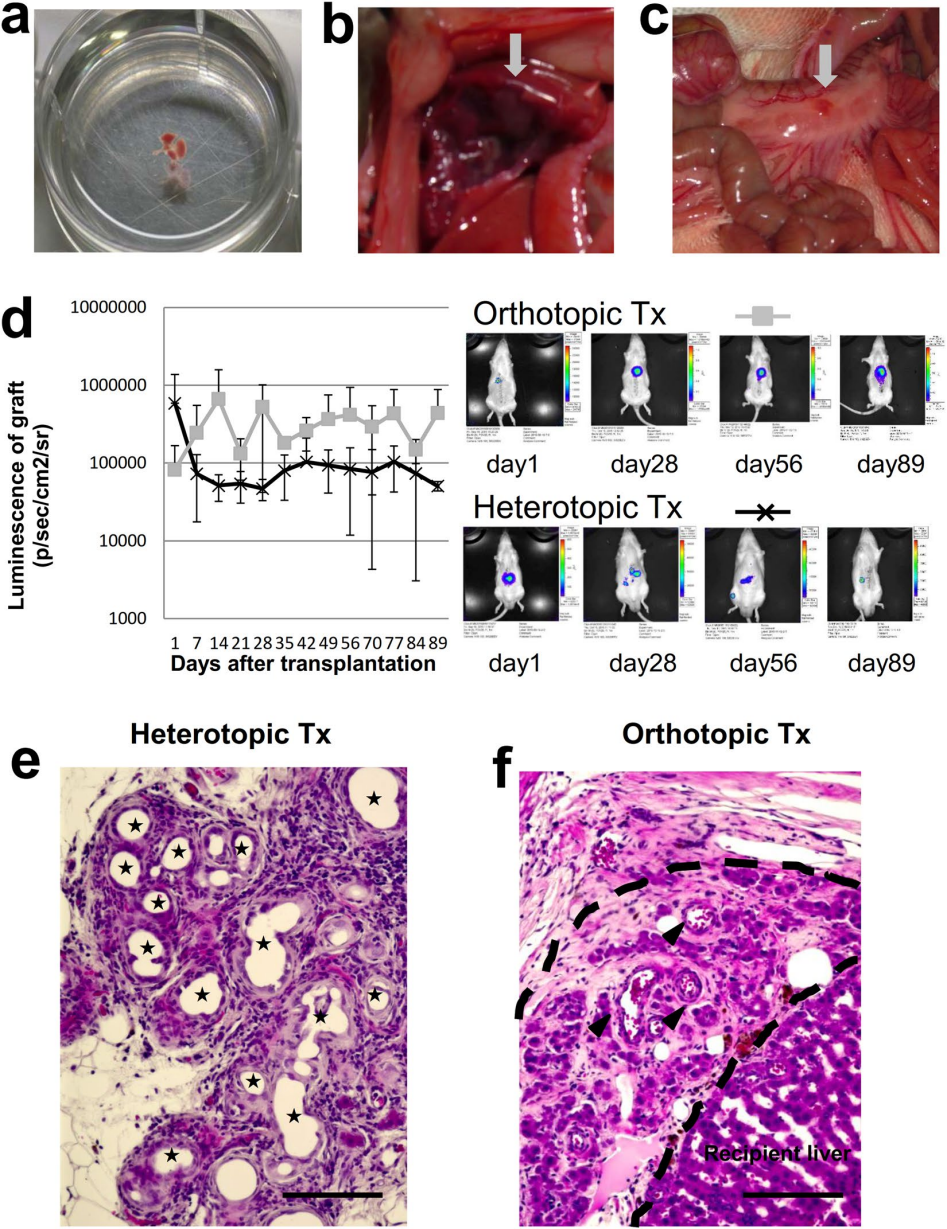
The transplantation of the liver bud on the transected parenchyma vs the mesentery. (**a**) A liver bud harvested from the fetus of a luciferase transgenic rat. (**b**) Orthotopic transplantation on the transected parenchyma of the liver. (**c**) Heterotopic transplantation into the proximal mesenterium. The grey arrow indicates the transplanted liver bud. (**d**) *In vivo* bioluminescence imaging of the grafts following orthotropic and heterotopic transplantation. Orthotopic transplantation: gray square, n = 3. Heterotophic transplantation: black cross, n = 4. The results represent the mean ± SD. (**e,f**) An HE-stained cross-section of the transplanted liver bud on the 90^th^ day after (**e**) heterotopic and (**f**) orthotopic transplantation. (**e**) The stars indicate pseudoglandular structures. (**f**) The dashed line indicates the transplanted liver bud. Mature hepatocyte-like cells were observed. The arrowheads indicate blood vessels. Scale bars, 100 μm.

Proximal mesenteric transplantation was the most efficient of the various heterotopic transplantation methods for transplanting *in vitro*-generated liver buds^20^. We compared transplantation onto the transected parenchyma with proximal mesenteric transplantation. Liver buds harvested from the fetus of a luciferase transgenic rat were used (Fig. 4a–c). For the first 7 days after transplantation into the proximal mesenterium, the luminescence of the graft decreased; thereafter, it was maintained at a lower level. In contrast, after transplantation onto the transected parenchyma, the luminescence of the graft increased for the first 14 days and was then maintained at a higher level for 3 months. The luminescence after transplantation onto the transected parenchyma was higher from the 7^th^ day and was significantly higher (1.83 × 10^6^ ± 2.77 × 10^4^ vs 8.03 × 10^4^ ± 4.70 × 10^4^, *p = 0.02*) on the 35^th^ day after transplantation (Fig. 4d). On the 90^th^ day after transplantation, a histological analysis showed that after transplantation into the proximal mesenterium, pseudoglandular structures were abundantly induced in the transplanted liver bud, indicating the suppression of liver development (Fig. 4e)^21,22^. In contrast, after transplantation onto the transected parenchyma, mature hepatocyte-like cells were observed (Fig. 4f). This result proved that orthotropic transplantation provided a more hospitable environment for the growth of the transplanted liver bud.

### The mass production of liver bud-like spheroids optimized for 3D printing

The unarranged fusion of spheroids could not to form controlled or uniform structures. Furthermore, the spheroids did not fuse at the central area due to the lack of medium supply in static culture conditions (Supplementary Fig. 1). To overcome these problems, we used a new 3D printing technology to create a reproducible structure and enable culture circulation.

In our preliminary experiments, we investigated the optimal shape of spheroids for the3D bio-printer. The diameter of 500–600 μm was required not to be sucked by the nozzle of the robotic arm. The shape needed to be as round as possible in order that the needle could skewer at the center of a spheroid. ≥60% roundness, which reflects the circularity of the shape, was the reference point. Although previous reports highlighted that self-organization through interaction with mixed cells was important for generating liver buds *in vitro*^5,7,23^; the reproducibility of this method in generating uniform structures was not validated. Thus, we first established the appropriate culture conditions for generating a large number of liver bud-like spheroids (LBSs) of uniform shape to be able to perform 3D printing stably, which contained human mature hepatocytes (hHeps), human umbilical vein endothelial cells (HUVECs) and human mesenchymal stem cells (hMSCs) on U-bottomed, 96-well plates with a low attachment surface.

**Figure 5:**
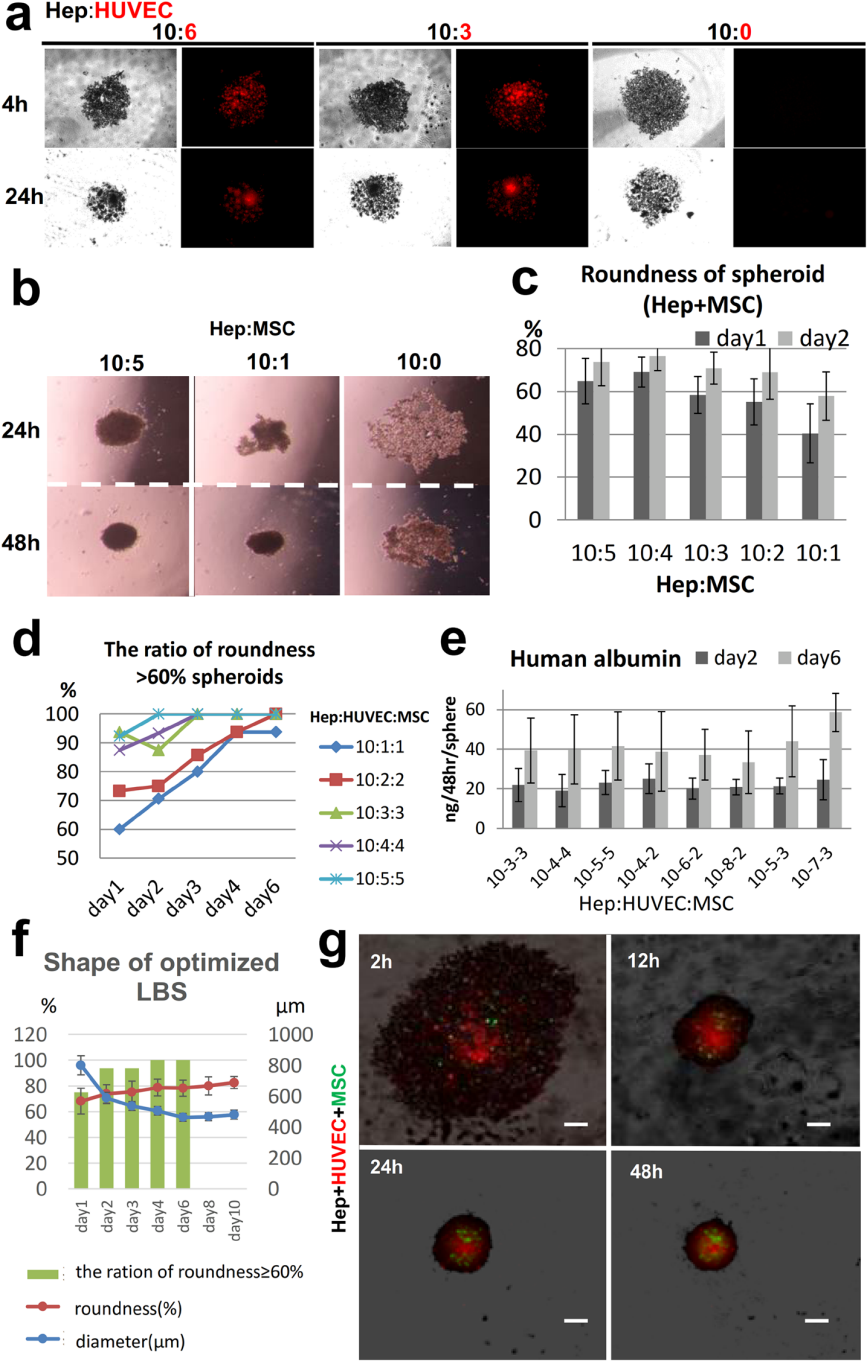
The establishment of appropriate culture conditions for generating a large number of liver bud-like spheroids. (**a**) Microscopic observation of spheroid formation at various ratios of hHeps and HUVECs. hHeps, black; HUVECs, red. (**b,c**) The morphological analysis of heterospheroid formation at various ratios of hHeps and hMSCs. (**b**) The microscopic observation of spheroid formation. The addition of hMSCs dramatically promoted hepatocyte aggregation and a single spheroid was generated in 2 days. (**c**) The roundness of the heterospheroid at various ratios of hHeps and hMSCs. The results represent mean ± SD; n = 12. (**d**) The ratios of the spheroids that had a roundness of ≥ 60% at various ratios of hHeps, HUVECs and hMSCs; n = 16. (**e**) The secretion of albumin into the culture medium was measured by an ELISA. LBSs containing hHeps, HUVECs and hMSCs at a ratio of 10:7:3 showed the significantly highest level of albumin secretion. The results represent the mean ± SD; n = 8. (**f**) The profiles of the optimized spheroid (hHeps:HUVECs:hMSCs at 10 × 10^3^:7 × 10^3^:3 × 10^3^ cells). Changes in the diameters and roundness of the spheroids and the ratios of the spheroids that had the roundness of ≥ 60%. The results represent the mean ± SD; n = 16. (**g**) The microscopic observation of spheroid formation using mixtures of the three cells types (hHeps: black; HUVECs: red; and MSCs: green). Scale bar, 300 μm.

Although the individual cultures of cryopreserved human hepatocytes did not form a single spheroid, they formed numerous small aggregates. When HUVECs were added, although the HUVECs could self-assemble, they had little effect on hepatocyte aggregation (Fig. 5a). As shown in previous reports, the addition of hMSCs promoted aggressive hepatocyte aggregation^23,24^ and a single spheroid was generated after 2 days (Fig. 5b). The measurement of the roundness of a spheroid showed that roundness increased as the number of hMSCs increased, in a time-dependent manner due to aggregation (Fig. 5c). Next, we analyzed spheroids with a roundness of value ≥ 60%, with various ratios of hHeps, HUVECs and hMSCs. The roundness of the spheroids increased as the number of stromal cells (HUVECs and hMSCs) increased. Approximately 90% of the spheroids exceeded this reference point on day 2, when the proportion of stromal cells was >37.5% (Fig. 5d). Finally, the secretion of human albumin in the culture medium was compared among the spheroids containing >37.5% stromal cells (at various ratios). The level was observed in LBSs containing hHeps, HUVECs, and hMSCs at a ratio of 10:7:3 was highest (to a significant extent; Fig. 5e). Finaly the optimized ratio of hHeps, HUVECs and hMSCs was found to be 10 × 10^3^:7 × 10^3^:3 × 10^3^ cells/spheroid that fulfilled the reference points of the diameter and roundness (Fig. 5f). Microscopic observation of spheroid formation showed that the HUVECs assembled quickly and were mainly localized at the central regions. hHeps assembled slowly by interacting with MSCs and were located at the circumference. MSCs were homogenously distributed (Fig. 5g).

### *The ex vivo* fabrication of a liver-like tissue using a new 3D bioprinting technology

**Figure 6:**
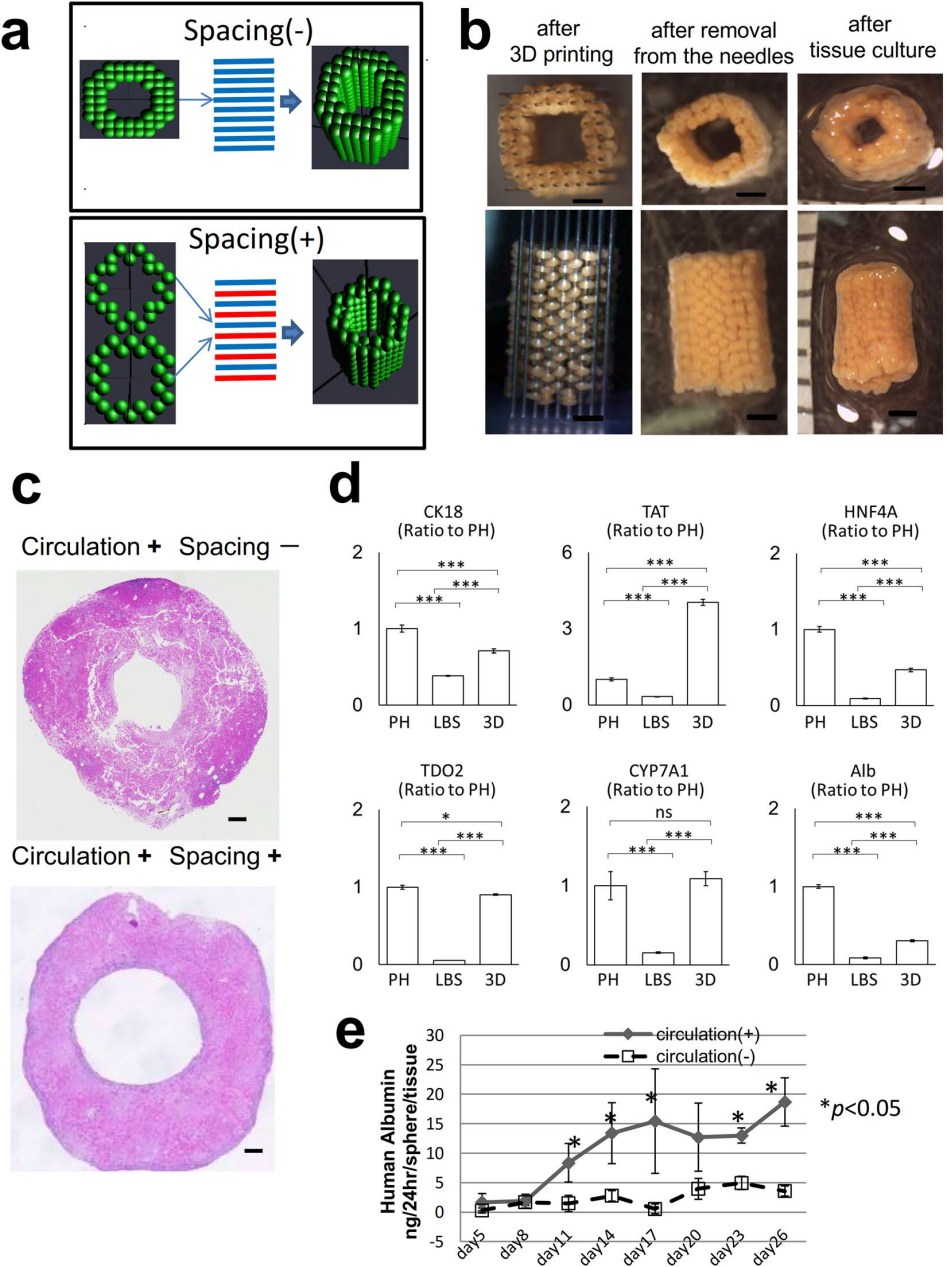
The construction of a scalable liver-like tissue using the new 3D bioprinter. (**a**) Comparative designs of the tube-shaped construct with or without spacing. (**b**) Morphological changes during 3D tissue construction. Scale bar, 200 μm. (**c**) The comparison of the effects of the spacing in HE-stained cross-sections on the 7^th^ day of culture. (**d**) Real-time RT-PCR analysis of the hepatic gene expression profiles mRNA in primary hepatocyte (PH), liver bud-like spheroid (LBSs) at the 2^nd^ day in culture, and 3D liver tissue at the 5^th^ day in culture (the day of transplantation). n = 3 for all groups. *P < 0.05, **P < 0.01, and ***P < 0.005. ns: no significance. Graph bars show the means ± SD. Cytokeratin 18:CK18, tyrosine aminotransferase: TAT, Hepatocyte nuclear factor 4 alpha: HNF4a, Tdo2 tryptophan 2,3-dioxygenase: TDO2, Cholesterol 7 alpha-hydroxylase: CYP7A1, albumin: Alb (**e**) The comparison of the secretion of albumin from the biofabricated tissue with/without culture circulation, as measured by an ELISA. Circulation (+), n = 4; circulation (−), n = 3. The results represent the mean ± SD. *p < 0.05.

The strategy used for the scaffold-free construction of 3D tissue with our 3D bioprinter was as follows: spheroids were assembled into a 3D shape on the needles of a pinholder (the needle-array system) using a robotic system according to the pre-designed 3D data. After the spheroids fused with each other, the needle-array was removed, and scaffold-free 3D tissue was obtained. (Supplementary Fig. 2a). On the 2^nd^ day of culture, we bio-printed LBSs to fabricate a tube-shaped tissue that could be perfused through the lumen to supply fresh medium from the inner space of the structure (Fig. 6a and Supplementary Fig. 2b,c).

The LBSs were fused in 3 days and were removed from the needle array. After 2 days of continuous culture, the holes from the needles were closed, resulting in slight shrinkage, and a scalable 3D structure was obtained (Fig. 6b). The size of a tube-shaped tissue specimen constructed with approximately 500 LBSs was 4 × 4 × 5mm.

Nutrients and oxygen were important for the construction of the 3D tissue^25,26^. We examined the influence of the spacing between each of the LBSs on the supply of culture medium (Fig. 6a) and the effects of culture circulation. We compared the histological findings of structures with or without spacing. On the 10^th^ day of culture, a histological examination of the structures without spacing showed cell death and tissue collapse. In contrast, the structure that was cultured with spacing and circulation showed the progression of spheroid fusion without collapse (Fig. 6c). Observation of several hepatic gene expression files (CK18, tyrosine aminotransferase: TAT, HNF4a, tryptophan 2,3-dioxygenase: TDO2, Cholesterol 7 alpha-hydroxylase and albumin) showed that although the functions of the hepatocyte decreased during spheroid formation with the significant degree, they significantly increased after 3D culture with the medium circulation. TDO2 recovered to the extent of primary hepatocyte and TAT achieved a four-fold expression of primary hepatocyte (Fig. 6d). Consequently, the comparison of the effects of culture circulation revealed that the level of albumin secreted by the tissue with circulation was significantly increased and that it was maintained at a high level for 1 month (Fig. 6d). The e*x vivo-*fabricated liver tissue was strong enough to tolerate physical manipulation (Supplementary Video 2).

### The self-organization of liver-like tissue after 3D printing

**Figure 7:**
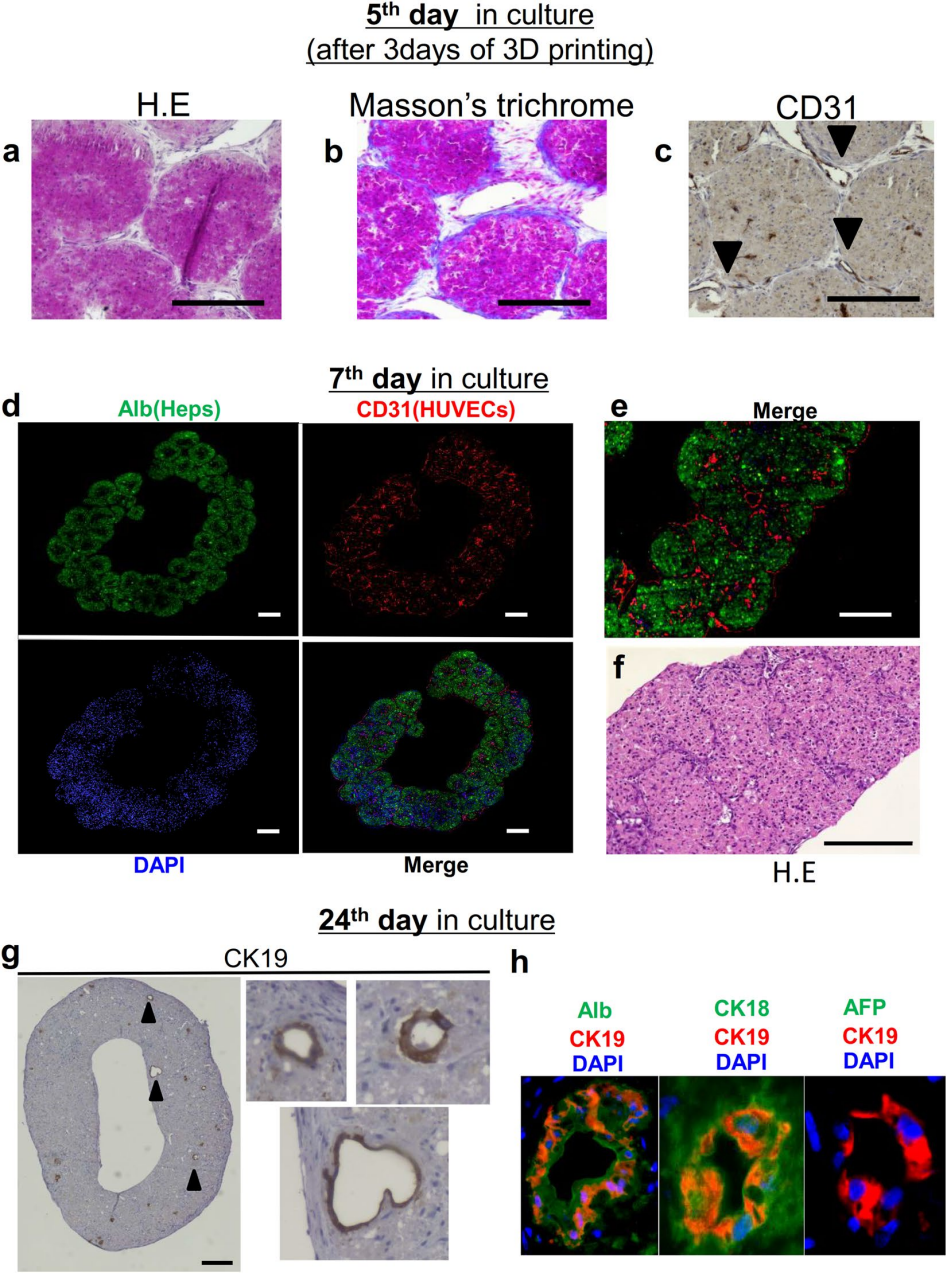
The histological analysis of *ex vivo*-fabricated liver-like tissue. (**a,b,c**) The histological analysis of the structures on the needles (3days after 3D printing) by hematoxylin-eosin staining, Masson’s trichrome staining and immunostaining using anti-human CD31 antibodies. (**b**) During tissue construction, the cells self-produced ECM. (**c**) HUVECs (indicated by the arrowheads) covered the surface of the tissue. (**d,e**) Fluorescence images of the liver-like tissue on the 7^th^ day in culture. hHeps were stained with anti-human albumin antibodies (green) and HUVECs were stained with anti-human CD31 antibodies (red). The production of albumin by hHeps and the formation of reticular endothelial networks inside the liver-like tissue were verified. Scale bars, 200 μm. (**f**) An HE-stained cross-section on the 7^th^ day in culture. The hepatocytes were viable, even in the center of the tissue, the thickness of which was approximately 600 μm. Scale bars, 100 μm. (**g**) Immunostaining of the liver-like tissue on the 24^th^ day in culture. CK19-positive cells and a duct-like morphology were observed. The arrowheads indicate the CK19-positive cells with a duct-like morphology. Scale bars, 200 μm. (**h**) Fluorescence images of the bile duct-like structure in the liver-like tissue.

At 3 days after 3D printing (on the 5^th^ day in culture), a histological analysis of the structure on the needle-array showed that the LBSs were fused together by direct attachment and connections with the extracellular matrix (ECM) (Fig. 7a,b). HUVECs tended to cover the surface of the structure along the ECM (Fig. 7c). On the 7^th^ day in culture, immunofluorescence microscopy revealed the production of albumin by hHeps and the formation of a reticular network by the HUVECs (Fig. 7d,e). H-E staining of the structure showed that the hHeps were viable, even in the deep area of the tissue (Fig. 7f). After 24 days of culture circulation, CK19-positive cells were observed in the 3D structure—some of them formed a duct-like structure (Fig. 7g). CK19-positive cells were also positive for CK 18 and human albumin, but negative for alpha fetoprotein (AFP) by immunofluorescence (Fig. 7h). These findings indicated that the primary hepatocyte transdifferetiated into the bile duct-like cells as shown in previous reports^27^. Conclusively, the 3D structure was growing into liver-like tissue through *ex vivo* tissue self-organization^28^.

### *The in vivo* engraftment of the biofabricated liver-like tissue on the transected liver parenchyma

**Figure 8:**
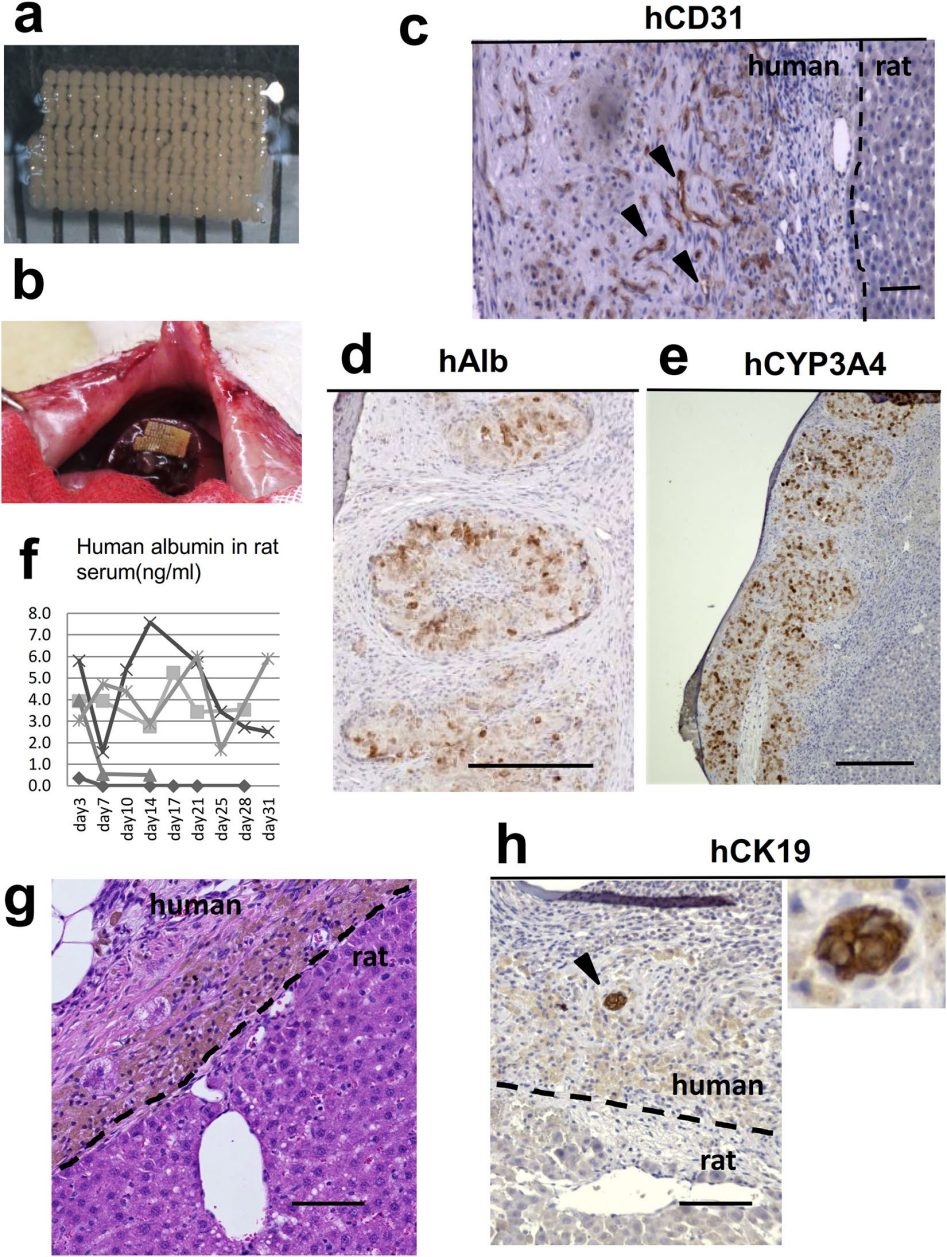
The transplantation of the *ex vivo*-fabricated liver-like tissue onto the transected liver parenchyma. (**a,b**) A transplanted liver-like tissue graft. (**c**–**e**) The histological analysis of the graft at 7 days after transplantation. (**c**) Vascular formation of HUVECs (indicated by arrowheads) was identified by the immunostaining of human CD31. The dotted line indicates the border of the rat liver. Scale bar, 100 μm. (**d**) The production of human albumin from the hHeps in the graft was detected by the immunostaining of human albumin. Scale bars, 200 μm. (**e**) The immunostaining of human CYP3A4 was positive in the graft. Scale bars, 200 μm. (**f**) The human albumin level in rat serum as measured by an ELISA. (**h**) An HE-stained cross-section on the 28^th^ day after transplantation. hHeps were observed within 100 μm from the recipient’s liver. Brown pigmentation was observed in the transplanted hepatocytes. The pigment was lipofuscin. Scale bar, 100 μm. (**g**) human CK19-positive cells (indicated by arrowheads) was identified by the immunostaining in the fibrous tissue of the graft. Scale bar, 100 μm.

Finally, to determine the transplantability of the biofabricated liver-like tissue, we transplanted a plate-shaped tissue specimen, which was constructed from one layer of approximately 200 LBSs, the size of which was 5 × 8mm (Fig. 8a), into nude rats using our new transplantation method (Fig. 8b). The histological analysis at 7 days after transplantation showed the vascular formation of the HUVECs via human CD31 immunostaining (Fig. 8c). The production of human albumin by the hHeps in the graft was detected at 7 days after transplantation by human albumin immunostaining (Fig. 8d). Human CYP3A4 was also positive in the hHeps in the graft (Fig. 8e). Human albumin was also detected for 1 month in the serum of 3/5 rats; however, the concentration was low (Fig. 8f). hHeps survived within 100 μm from recipient’s liver; however, lipofuscin pigmentation (confirmed by the Schmorl method: data not shown) was observed in the hHeps, and fibrous tissue was increased in the transplanted human liver-like tissue on the 28^th^ day after transplantation (Fig. 8g). This result indicated that the supply of blood was insufficient for the graft and the limitation of the thickness of tissue without prevascularization prior to transplantation. human CK 19 positive cells were observed in the fibrous area of the transplanted human liver-like tissue (Fig. 8h). This result indicated that the bile ductular proliferation was occurred due to transplanted liver tissue damage; however, this also indicated that the biofabricated liver-like tissue had the potential of the bile duct formation *in vivo*.

## Discussion

We herein report two novel methods for growing *in vitro*-generated liver buds *in vivo* and *ex vivo*: orthotopic transplantation on the transected liver parenchyma to directly connect the transplant with the native liver tissue; and the biofabrication of a scalable liver-like tissue through the 3D bioprinting of LBSs. Recently, a liver bud generated *in vitro* was shown to have therapeutic potential in mice^20^. The next challenge is to grow it to human scale *in vivo* and *ex vivo*. The liver is commonly known to be a regenerative organ with a predominant portal supply. Portal vein arterialization inhibits liver regeneration and causes the liver to shrink^29^. Considering that transplanted liver tissue at an ectopic site is supplied via an artery, orthotopic transplantation is feasible for achieving the growth of a liver bud. Our findings, that the luminescence increased after orthotopic transplantation and decreased after heterotopic transplantation, support this logical conclusion. Furthermore, we observed that pseudoglandular structures were abundantly induced in the transplanted liver bud following transplantation into the proximal mesenterium. In contrast, hepatic architecture was observed following transplantation onto the transected parenchyma. Pseudoglandular structures were clearly observed in the mouse liver, which had a defect regarding the control of hepatic growth^21,22^. Thus, our findings revealed that transplantation onto the transected parenchyma enabled the growth of the transplanted liver bud, while liver development was suppressed after transplantation into the mesenterium. In addition to the portal supply, it was hypothesized that the growth of the transplanted liver bud was achieved, not only through the regenerative signal from the local release of growth factors such as hepatocyte growth factor (HGF) and matrix remodeling, but also through the direct interactions between the hepatic cells in the graft and the hepatic cells in the recipient’s liver^30^. Collectively, the important feature of our transplant method is that it allows for direct connection between the transplant and the native liver tissue through the creation of an intact parenchyma. Operability was even confirmed in micro mini pigs (Supplementary Fig. 3). Since avoiding coagulation hemostasis increases the risk of bile leak and bleeding from the transection plane^31,32^, further studies are needed to investigate the techniques of hepatic transection and the materials for covering the transection plane.

There are several approaches to constructing a scalable 3D cellular structure using spheroids as building blocks. Some studies have constructed liver tissue using a scaffold^15^. A scaffold-free approach can avoid several problems that are associated with the use of exogenous materials in scaffold-based tissue engineering approaches, including infection, immune reactions, and the degradation of materials and biofilms^16^. Since our technology uses needles made from medical grade stainless steel to fix the spheroids and because exogenous materials are not used at any stage of the fabrication process, the constructed tissue is not contaminated by foreign materials, which implies its clinical safety. Another advantage of our technology is that it enables the fabrication of elaborate geometry in 3D tissue and the fixation of the shape with the needle-array system during the period of spheroid fusion. A previous method of constructing scaffold-free structures using spheroids used molds to fix the spheroids^14^. The use of mold to maintain the geometry of the assembled spheroids^33^ would likely make it difficult to construct a stable structure with culture circulation. In the present study, we fabricated a tube-shaped tissue to perfuse the culture medium into the 3D structure. When we fabricated a solid 3D structure and performed the culture circulation around it, the supply of the culture medium into the 3D structure was poor. Therefore, we fabricated a tube-shaped structure to supply fresh medium from the central lumen. The spaces for supplying medium into the structure also worked to avoid an ischemic environment *ex vivo*. To construct this elaborate geometry, the controlled formation of spheroids of uniform shape was required. Thus, we established the culture conditions of the LBSs of uniform shape.

Our technology was limited by the thickness of the spheroids that could be stably manipulated by our 3D bioprinter. The LBSs fabricated here had a diameter of approximately 500 μm. If hepatocyte spheroids were to be used *in vitro*, the viable diameter would be limited to approximately 100–150 μm due to the depletion of oxygen^34–36^. In this respect, using a mixture of multiple cells offered several benefits. We observed that hHeps were located at the circumference, while HUVECs and MSCs were located at the core. This distribution was expected to alleviate the hypoxic condition of the hHeps. Furthermore, a previous study reported that the combination of HUVECs and MSCs promoted vascular formation through the release of vascular endothelial growth factor (VEGF) and HGF under hypoxic conditions^37,38^. However, observation of the hepatic gene expression files by RT-PCR showed that the hepatic function of the LBSs were down-regulated early after aggregation. In this study, we mixed the three cells in different lineage stage: mature hepatocytes and the mesenchymal cells in early lineage stage: HUVECs and MSCs. The formation of cell aggregates and self-organization naturally require lineage dependent interactions and the cooperative maturation. Therefore, the usage of hepatic progenitor cells on behalf of mature hepatocytes, or the late lineage stage endothelia and stellate cells on behalf of HUVECs and MSCs may improve these results. The human albumin levels in liver tissue transplanted rats were very low. That was also due to the down-regulation of the hepatic function in the spheroid culture. Although we should optimize the culture condition such as the population of the cells in the multicellular spheroids, we emphasize the benefit from the 3D culture using our 3D bio-printer on the hepatic function as shown in the RT-PCR (Fig. 6d) and the ELISA (Fig. 6e). In this study, we transplanted the biofabricated tissues as soon as the LBSs fused each other in order to investigate the growth of the transplant organoid. According to these results, the transplantation at the later day of culture would improve the functional results *in vivo*.

The biofabricted liver-like tissues survived within 100 μm after transplantation on rat liver—which was equivalent to the maximum thickness of the prevascularized multi-layered cell-sheet^25^. Considering the findings regarding the limitation of the thickness of the transplanted tissue (which was only supplied from the bottom side), it may be useful to fabricate vascularized tissue before transplantation to supply blood flow to the distal area. Early vascularization after transplantation is an important challenge in the tissue transplantation. After 1 month from transplantation, the transplanted liver-like tissue was not healthy and covered by the fibrotic plaque. The reason was poor survival of vessels in the graft. That was possibly due to poor vascularization from the native liver. Another possible reason was rejection. Even though a nude rat is an immunocompromised animal, it possesses a few B cells, Natural Killer cells and macrophages. Furthermore, the fibrotic tissue might be induced by the collagen sheet attached on the stump of the liver. The transplantation of the biofabricated liver tissue without covering by collagen sheet, in which we directly anastomosed the biofabricated liver tissue on the transected parenchyma of liver and covered with omentum, decreased the fibrotic plaque (Supplementary Fig. 4). The choice of the material to cover the liver tissue graft will be the next challenge.

Although the biofabricated liver-like tissue constructed with mature hepatocytes showed the potential to form bile duct-like structures in the present study, the tissue should be fabricated from hepatic progenitor cells that have the potential to differentiate into both hepatocytes and cholangiocytes^39–41^ in order to facilitate bile duct formation, and also to match the lineage stage of the mixed cells as discussed above. The functional bile duct formation should be one of the important advantages of the orthotopic transplantation.

In summary, our transplantation method has two major advantages: it does not involve any vascular obstructions and the presence of direct connection between the graft and the recipient’s liver parenchyma would facilitate greater graft growth. Our 3D bioprinting technology has two main advantages: it allows for the fabrication of elaborate geometry in 3D tissue and the fixation of the shape with the needle-array system enables the immediate execution of culture circulation after 3D printing—thereby avoiding an ischemic environment *ex vivo*—which supports the biofabrication of scalable tissue. Although the effort to improve the optimal culture condition of multicellular spheroids and 3D tissue, future studies of engineering of organoid, especially prepared from stem cell, will facilitate tissue-based replacement therapy in the liver using the methods described in this study.

## Methods

### Animal experiments

All of the animal experiments were approved by the Institutional Animal Care and Use Committee of Kyushu University (Protocol Numbers: A27-122-1, A26-207-2) and Keio University and also conformed to all of the guidelines outlined in the Guide for the Care and Use of Laboratory Animals by the National Institutes of Health (NIH). All laparotomies in rats were performed after the intraperitoneal injection of medetomidine (0.15 mg/kg) + midazolam (2 mg/kg) + butorphanol tartrate (2.5 mg/kg) or under anesthesia with 2% isoflurane. The animals were euthanized with the inhalation of isoflurane and an overdose of pentobarbital.

### The creation of an intact transection plane on the liver without thermal injury

After laparotomy in a recipient rat, the left median lobe was slowly bound below the mid-transverse line with a 4-0 silk suture. During the tightening of the suture, the liver parenchyma was bluntly resected and the blood vessels and bile duct of the median lobe were ligated. If oozing bleeding was observed from the transection plane, pressure was applied to achieve hemostasis. The liver tissue graft was attached on the transected parenchyma and capped with a collagen sheet (Collagen Vitrigel, AGC Techno Glass, Shizuoka, Japan) (Fig. 3 and see also Supplementary Video 1).

### The comparison of the transplant sites

To access the intact liver parenchyma, we devised two orthotropic transplant methods that avoided thermal injury. One method was intra-liver transplantation into a fissure on the liver surface that was created by blunt dissection; the other was transplantation on the transection plane. We initially compared these two methods. The recipient rats were wild-type Lewis rats (250–300 g); the donor rats were luciferase-transgenic Lewis rats^19^. A small chip of the liver tissue harvested from the donor rat was transplanted via intra-liver transplantation or transplantation on the transection plane with left lateral lobectomy (30% partial hepatectomy). Next, we compared transplantation onto the transected parenchyma to proximal mesenteric transplantation. We transplanted liver buds harvested from the fetus of a luciferase-transgenic rat (E13.5). For transplantation in the proximal mesentery, the graft was placed under the serous membrane of the mesentery. For growth stimulation, left lateral lobectomy was performed during the transplant operation.

### Cell preparation

HUVECs and hMSCs (Lonza, Basel, Switzerland) were purchased and subcultured in endothelial growth medium (EGM, Lonza) or MSC growth medium (MSCGM, Lonza) at 37 °C in a humidified 5% CO_2_ incubator and passaged until the required numbers of cells were obtained. Cryopreserved human platable hepatocytes (hHeps) (Life Technologies, Carlsbad, CA, USA; Lots HU 1420 and 8199) were purchased and stored in liquid nitrogen. Human hepatocytes were used for the spheroid culture immediately after thawing.

### Spheroid culture

Spheroids were generated in each well of U-bottomed, 96-well plates with a low attachment surface (Thermo Fisher Scientific, Waltham, MA, USA). hHeps were thawed, and the number of viable cells was counted according to the manufacturer’s instructions. The viable cells (85–95%) were used. hHeps and hMSCs were mixed at various ratios in 200 μl of hepatocyte plating medium (William’s E medium supplemented with 1 μM dexamethasone [Life Technologies], Plating Cocktail A [Life technologies], 10 mM nicotinamide [Sigma-Aldrich, St. Louis, MO, USA], 10 ng/ml human recombinant hepatocyte growth factor [HGF, Sigma] and 10% fetal bovine serum [FBS, Sigma]). hHeps and HUVECs were mixed at various ratios in 200 μl of the spheroid formation medium (hepatocyte plating medium further supplemented with hFGF-B, VEGF, R3-IGF-1, heparin, ascorbic acid, and GA-1000 from an EGM2 Bullet Kit [Lonza]). The combination of the three cells was analyzed at various ratios in 200 μl of the spheroid formation medium. The spheroids were maintained at 37 °C in a humidified 5% CO_2_ incubator. The co-culture medium was replaced every 2 days, and the spent media were used for the subsequent analysis.

### The scaffold-free construction of scalable liver tissue using a 3D bioprinter

Three-dimensional tissue was constructed using a 3D bioprinter (Regenova. Cyfuse Biomedical K.K., Tokyo, Japan). The strategy used for the scaffold-free construction of 3D tissue was as follows: spheroids were assembled into a three-dimensional shape on the needles of a pinholder (the needle-array system) using a robotic system according to the pre-designed 3D data. After the spheroids fused with each other, the needle-array was removed, and scaffold-free 3D tissue was obtained (Supplementary Fig. 2a). The spheroids were picked up with a suction nozzle (O.D of 0.45 mm and I.D. of 0.23 mm) and skewered onto needles made from medical-grade stainless steel, which were 170 μm in diameter and arrayed 400 μm apart, according to the 3D design data. After 3D printing, we cultured the structure in the maintenance medium (William’s E medium supplemented with 0.1 μM dexamethasone (Life Technologies), Cell Maintenance Cocktail B (Life Technologies), 10 mM nicotinamide (Sigma-Aldrich), 10 ng/ml HGF (Sigma) and hFGF-B, VEGF, R3-IGF-1, heparin, ascorbic acid, and GA-1000 from an EGM2 Bullet Kit (Lonza). The experimental protocol and the timetable for constructing the liver tissue *in vitro* are shown in Supplementary Fig. 2b. To evaluate the effect of spacing between each of the LBSs to supply culture medium, we fabricated a tube-shaped tissue with or without spacing (Fig. 6a).

### The circulation chamber and culture circulation

The circulation chamber and tissue culture system are shown in Supplementary Fig. 2c. The structures were placed in a polycarbonate culture box (Asahi Glass, Tokyo, Japan) with inflow and outflow ducts. The culture medium was perfused by a roller pump at a flow rate of 2 ml/min. The tube-shaped structures on the needle array were cultured in a maintenance medium that was perfused through the lumen of the needle array, which was connected to the inflow duct. After removal from the needle array, cultivation was continued on an 18-gauge IV cannula (Terumo, Tokyo, Japan) with side slits was continued to perfuse the medium into the lumen. The tissue culture was maintained at 37 °C in a humidified 5% CO_2_ incubator. The volume of the culture medium was adjusted to 300 μl/spheroid and the medium was replaced every 3 days; spent media were used for the subsequent analysis.

### The transplantation of *ex vivo-*fabricated 3D liver tissue onto the transected liver parenchyma

The plate-shaped tissue was fabricated on one layer of needles on a large size pinholder with a central lumen. After 5 days of cultivation (2 days of spheroid formation and 3 days of cultivation on needles), the liver-like tissue was transplanted onto the transection plane of the liver in nude rats (F344/NJcl-rnu/rnu, 200–250 g, male, CLEA Japan, Tokyo, Japan). For the functional analysis of the transplanted liver tissue, blood samples were collected to measure the amount of human-specific albumin that was produced in the recipient rats. For the histological analysis, the livers of the recipient rats were removed, and the animals were euthanized.

### Bioluminescence imaging

The viability of the graft was tracked using the IVIS200 bioluminescence imaging system (Perkin Elmer, Waltham, MA, USA) after the intraperitoneal injection of 3 ml of a 30 mg/ml luciferin substrate under anesthesia with 2% isoflurane.

### The morphological analyses

Microscopic images of the spheroids were captured with a photomicroscope (CKX41SF, Olympus, Tokyo, Japan). Phase-contrast and fluorescence images were captured with a fluorescence microscope (BZ-700, Keyence, Okayama, Japan). For the fluorescence images, HUVECS were stained red with a fluorescent probe (Qtracker 605, Thermo Fisher Scientific) according to the manufacturer’s instructions. Stereoscopic images of the 3D structures were obtained with a stereo microscope (SZX7, Olympus, Tokyo, Japan).

### The measurement of the spheroid diameter and roundness

The spheroid diameters and roundness were measured using an on-board function of the 3D bioprinter. The formula used to calculate roundness was as follows: Roundness (%) = 100 − (R−r)/R × 100, where R = the radius of the minimum circumscribed circle and r = the radius of the maximum inscribed concentric circle. The measurement was performed on the first 2 days, then every 2 or 3 days.

### Histochemistry, immunohistochemistry and the immunofluorescence analysis

For the cross-sectional observations, tissues were fixed in 4% paraformaldehyde, processed, and embedded in paraffin. HE staining and Masson’s trichrome staining were performed using conventional methods. The following primary antibodies purchased from Abcam, Cambridge, UK were used. A monoclonal mouse anti-human serum albumin antibody (ab7793), a polyclonal rabbit anti-human CD31 antibody (ab28364), a monoclonal mouse anti-human CK18 antibody (ab82254), a monoclonal rabbit anti-human CK19 antibody(ab76539), a monoclonal rabbit anti-alpha Fetoprotein antibody (ab169552) and a monoclonal rabbit anti-human CYP3A4 antibody (ab124921). For immunohistochemistry, HRP detection was performed based on the micropolymer chemistry of the ImmPRESS™ Reagent (Vector Laboratories, Burlingame, CA, USA). FITC-conjugated goat anti-mouse IgG (H + L) (Invitrogen) and Alexa Fluor 555-conjugated goat anti-rabbit IgG (H + L) (Life Technologies;) or Opal 4-Color Manual IHC kit (Perkin Elmer, Waltham, MA, USA) were used for the immunofluorescence staining. Images were captured using a fluorescence microscope (BZ-X700, Keyence) and a confocal laser-scanning microscope (FV10i, Olympus).

### Quantitative real-time reverse transcript-polymerase chain reaction (RT-PCR) assay

The gene expression of primary hepatocyte alone and hepatocyte in LBS and 3D tissue were measured using qRT-PCR. Primary hepatocytes were analyzed immediately after thawing. After 2 days of spheroid formation culture, LBSs were collected into Eppendorf tubes and then coryopreserved at −80 °C. At the 2^nd^ day of culture, the 3D-printing was performed and the tube-shaped 3D tissues were cultured for 3 days with the culture circulation. At the 5^th^ day of culture (the day of transplantation), constructed 3D tissues were collected into Eppendorf tubes and then coryopreserved at −80 °C. LBSs and 3D tissues were analyzed immediately after thawing.

Total RNA from cultured cells and tissue samples was prepared with TRIzol (Invitrogen) and DNase I (Promega, Madison, WI), and was purified using an RNeasy Mini Kit (Qiagen). The cDNA was prepared by a reverse transcription reaction using Revertra Ace qPCR kit (TOYOBO, Osaka, Japan). qRT-PCR was subsequently performed using a TaqMan Gene Expression Master Mix (Applied Biosystems, Foster City, CA) and target TaqMan probes (Applied Biosystems) or using a SYBR Premix Ex Taq II kit (Takara) with target specific primers.with a Light Cycler 96 (Roche, Indianapolis, IN). 18 S ribosomal RNA was used for normalization. The probes and primers for qRT-PCR were summarized in Supplementary Table 1.

### The measurement of albumin secretion

The levels of albumin secreted in the culture medium and blood samples were measured using a Human Albumin ELISA Quantitation Kit (Bethyl Laboratories, Montgomery, TX, USA) according to the manufacturer’s instructions.

### Statistical analysis

The statistical analyses were performed using the JMP^®^ 11 software program (SAS Institute Inc., Cary, NC, USA). The data are expressed as the mean ± SD. Comparisons between two groups were made using the unpaired Student’s *t*-test. P values of <0.05 were considered to indicate statistical significance.

## Electronic supplementary material

Supplementary Information

Orthotropic transplantation method on the transected parenchyma.

Physical strength of the ex vivo-fabricated liver tissue.
