# Supplementary material for: In vivo and ex vivo methods of growing a liver bud through tissue connection
Source: Sci Rep. 2017 Oct 26;7:14085. doi: 10.1038/s41598-017-14542-2 (PMC5658340; doi:10.1038/s41598-017-14542-2)
Supplement: Supplementary file 1 — Supplementary Information [file 41598_2017_14542_MOESM1_ESM.pdf]

Title: ***In vivo* and *ex vivo* methods of growing a liver bud through tissue connection**

Yusuke Yanagi[1], Koichi Nakayama[2**], Tomoaki Taguchi [1], Shin Enosawa[3], Tadashi Tamura[4], Koichiro Yoshimaru[1], Toshiharu Matsuura[1], Makoto Hayashida[1], Kenichi Kohashi[5], Yoshinao Oda[5], Takayoshi Yamaza[6], Eiji Kobayashi[3*]

[1] Department of Pediatric Surgery, Reproductive and Developmental Medicine, Kyushu University Graduate School of Medical Sciences, 3-1-1 Maidashi, Higashi-ku, Fukuoka 812-8582, Japan

[2] Department of Regenerative Medicine and Biomedical Engineering, Faculty of Medicine, Saga University, 1 Honjo-machi, Saga 840-8502, Japan

[3] Department of Organ Fabrication, Keio University School of Medicine, 35 Shinanomachi, Shinjuku-ku, Tokyo, 160-8582, Japan

[4] Cyfuse Biomedical K.K., 2-27-17Hongo, Bunkyo-ku, Tokyo, 113-0033, Japan

[5] Department of Anatomic Pathology, Pathological Sciences, Kyushu University Graduate School of Medical Sciences, 3-1-1 Maidashi, Higashi-ku, Fukuoka 812-8582, Japan

[6] Department of Molecular Cell Biology and Oral Anatomy, Kyushu University Graduate School of Dental Science, 3-1-1 Maidashi, Higashi-ku, Fukuoka 812-8582, Japan

Supplementary Information

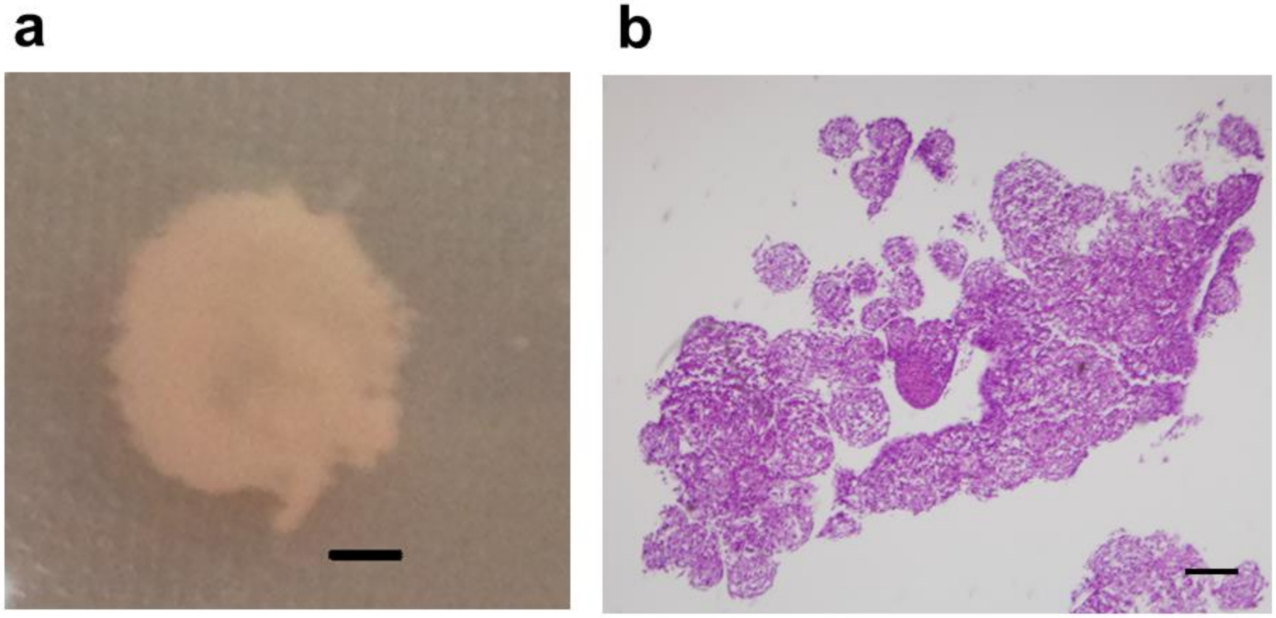

Supplementary Figure 1. Scalable tissue fabricated using the unarranged fusion of spheroids. (**a**) Gross observations of scalable tissue formed by the parallel fusion of small spheroids in a low attachment dish. Scale bar, 250 μm. (**b**) An HE-stained cross section of the tissue. Scale bar, 200 μm.

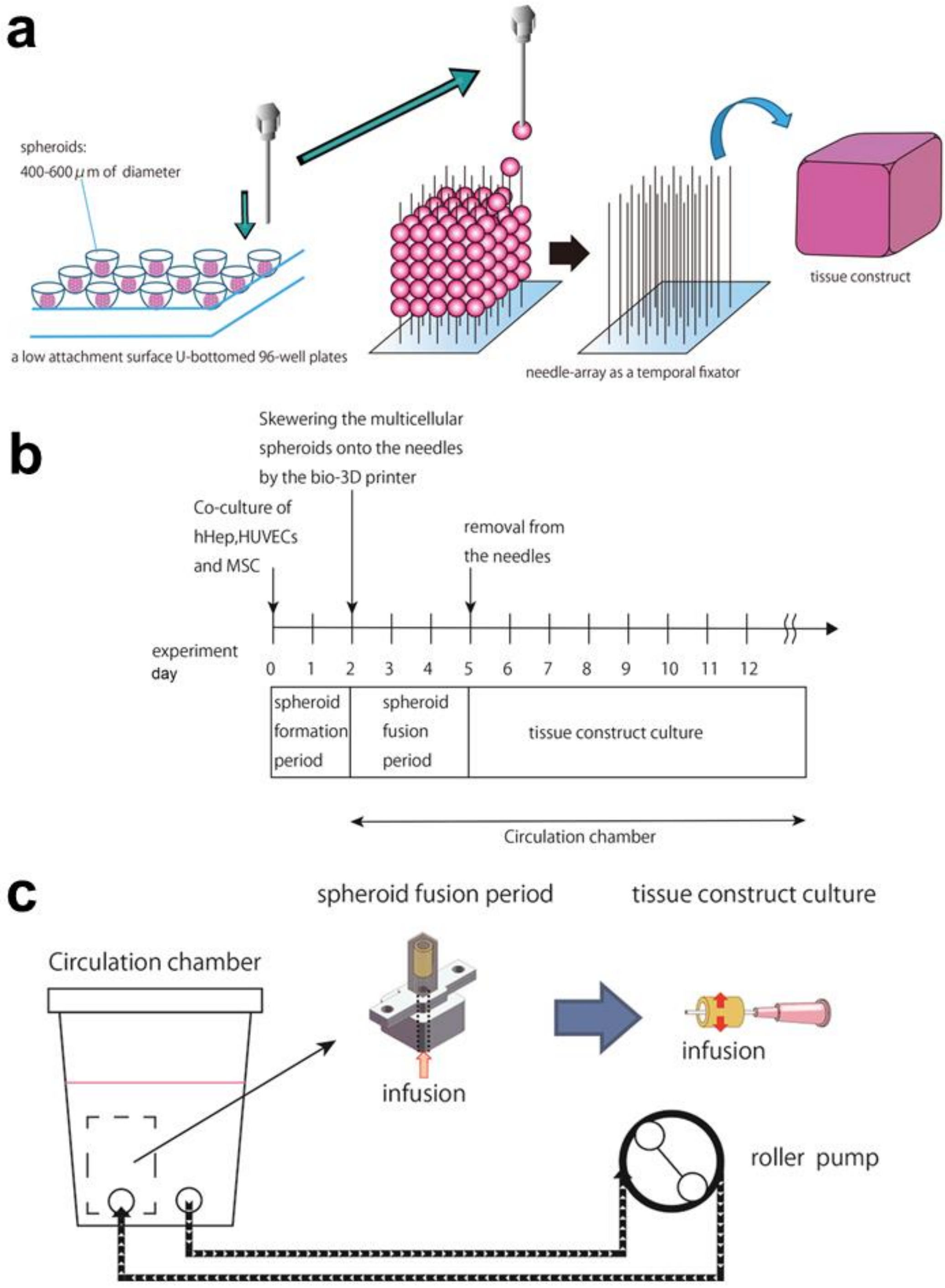

Supplementary Figure 2. The experimental strategy used to construct liver-like tissue with a new 3D bioprinter.

(**a**) A schematic representation of our 3D bioprinter. (**b**) The timetable for constructing the liver tissue from liver bud-like spheroids *in vitro*. (**c**) The circulation system for culturing 3D tissue.

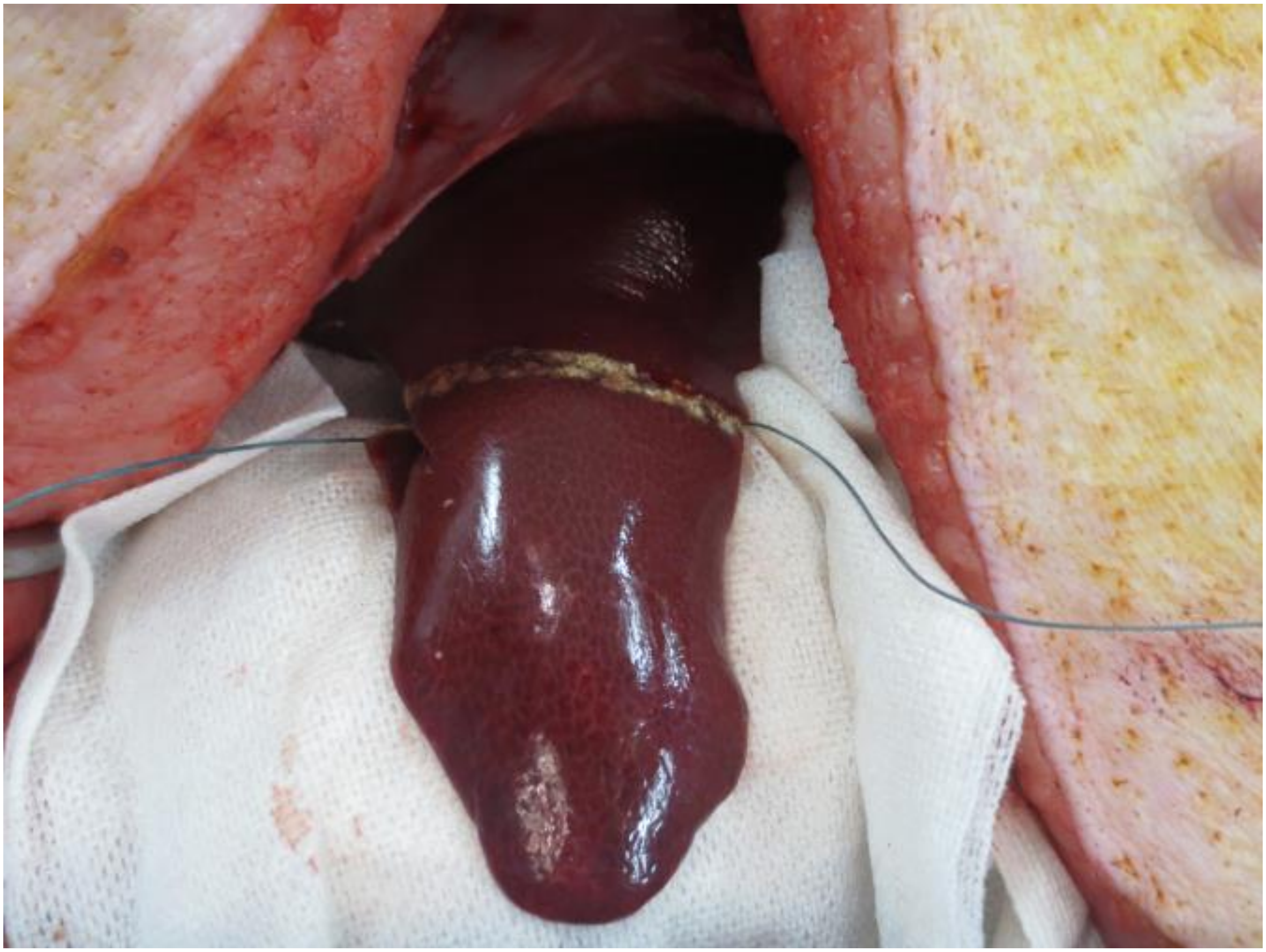

Supplementary Figure 3. The creation of an intact transection plane of the liver in a micro mini pig.

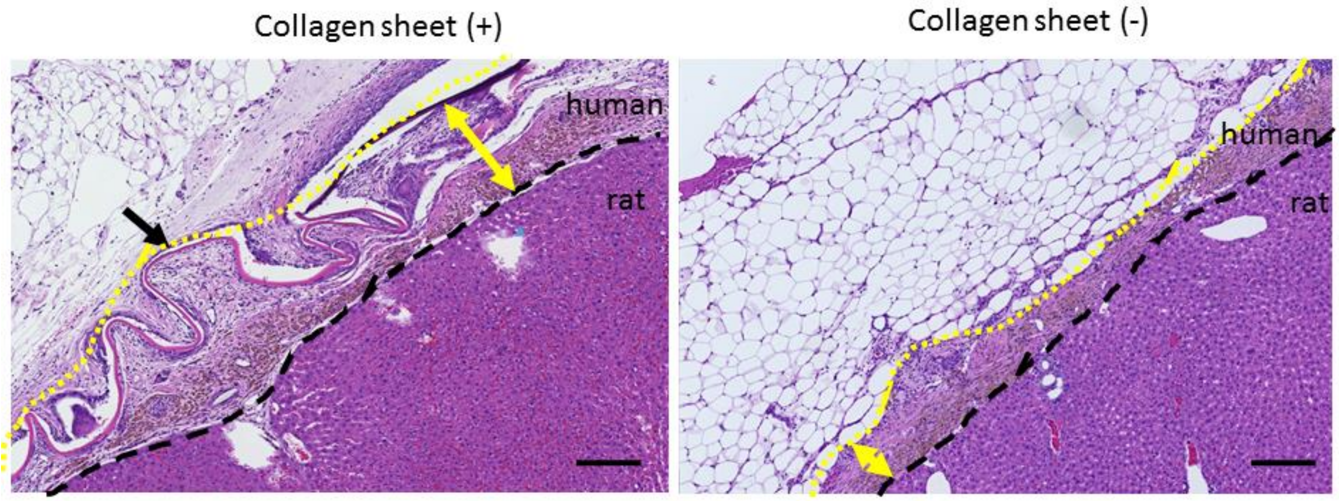

Supplementary Figure 4. Comparison of the fibrotic plaque surrounding the transplant with or without the covering with a collagen sheet.

An HE-stained cross-section on the 28th day after transplantation. The pink sheet indicated by the black arrow was the collagen sheet. The black dashed line indicates the border between the rat liver and the transplanted 3D liver tissue. The yellow dashed line indicates the border between the transplanted 3D liver tissue and omentum. The yellow two-way arrow indicates the thickness of the fibrotic plaque in the transplanted tissue. Scale bar, 100 μm.

.

Supplementary Table 1.
[TaqMan Probe]

18S; Eukaryotic 18S rRNA
Product ID: Hs99999901_s1

CK18; KRT18; keratin 18
Product ID: Hs02827483_g1

TAT
Product ID: Hs00356930_m1

HNF4A; hepatocyte nuclear factor 4 alpha
Product ID: Hs00230853_m1

ALB: albmin
Product ID: Hs00910225_m1

[SYBR Green Primer]

TDO2; tryptophan 2,3-dioxygenase
Forward：ggccatgtcagagatgaaagga
Reverse：gtccaaggctgtcatcgtct
Number：NM_005651.3
364～483→120bp

CYP7A1;　Cholesterol 7 alpha-hydroxylase
Forward：agtcagcttggaaggcaatc
Reverse：tcctccttagctgtccggat
Number：NM_000780.3
1038～1169→132bp

Supplementary Video 1. Orthotropic transplantation method on the transected parenchyma. An intact parenchyma at the transection plane was created by ligating and cutting the parenchyma with a surgical suture in a rat; hemostasis was achieved by the concurrent ligation of the vessels.

Supplementary Video 2. Physical strength of the ex vivo-fabricated liver tissue.

The e*x vivo*-fabricated liver tissue on the 7th day in culture was strong enough to tolerate physical manipulation
